# Combination treatment with rucaparib (Rubraca) and MDM2 inhibitors, Nutlin-3 and RG7388, has synergistic and dose reduction potential in ovarian cancer

**DOI:** 10.18632/oncotarget.19266

**Published:** 2017-07-15

**Authors:** Maryam Zanjirband, Nicola Curtin, Richard J. Edmondson, John Lunec

**Affiliations:** ^1^ Northern Institute for Cancer Research, Newcastle University, Newcastle Upon Tyne NE2 4HH, United Kingdom; ^2^ Faculty Institute for Cancer Sciences, University of Manchester, Manchester M13 9WL, United Kingdom

**Keywords:** ovarian cancer, targeted therapy, rucaparib, Nutlin-3/RG7388, combined treatment

## Abstract

Ovarian cancer is the seventh most common cancer worldwide for females and the most lethal of all gynecological malignancies. The treatment of ovarian cancer remains a challenge in spite of advances in debulking surgery and changes in both chemotherapy schedules and routes of administration. Cancer treatment has recently been improving with the introduction of targeted therapies to achieve greater specificity and less cytotoxicity. Both PARP inhibitors and MDM2-p53 binding antagonists are targeted therapeutic agents entered into clinical trials. This preclinical study evaluated the effect of Nutlin-3/RG7388 and rucaparib as single agents and in combination together in a panel of ovarian cancer cell lines. Median-drug-effect analysis showed Nutlin-3/RG7388 combination with rucaparib was additive to, or synergistic in a cell type-dependent manner. Mechanism studies showed rucaparib alone had no effect on p53 stabilization or activity. Although treatment with Nutlin-3 or RG7388 induced stabilization of p53 and upregulation of p21^WAF1^ and MDM2, the addition of rucaparib did not enhance the p53 activation seen with the MDM2 inhibitors alone. These results demonstrate that the synergistic effect on growth inhibition observed in the combination between rucaparib and Nutlin-3/RG7388 is not the result of increased p53 molecular pathway activation. Nevertheless, combined treatment of Nutlin-3/RG7388 with rucaparib increased cell cycle arrest and apoptosis, which was marked for A2780 and IGROV-1. These data indicate that combination treatment with MDM2 inhibitors and rucaparib has synergistic and dose reduction potential for the treatment of ovarian cancer, dependent on cell type.

## INTRODUCTION

Ovarian cancer is the fifth leading cause of cancer-related female deaths and was reported to be responsible for approximately 152,000 deaths worldwide in 2012 [1]. Although up to 80% of patients with primary disease respond to first-line chemotherapy, relapse and resistance to further treatment is prevalent, leading to lack of long-term benefit from treatment [2]. Mucinous and clear cell histological subtypes do not respond well to current chemotherapy strategies and are clinically challenging to treat [3, 4]. Different strategies have been developed to treat the recurrent and/or resistant disease, including targeted therapies such as angiogenesis inhibitors and poly(ADP-ribose) polymerase (PARP) inhibitors [5].

The inhibition of PARP enzymatic activity hinders DNA repair via the base excision repair (BER) pathway. For cells deficient in homologous DNA recombination repair (HRR) this results in multiple lethal DNA double-strand breaks normally repaired by the HRR pathway. The tumor cells with *BRCA1/2* mutation or other HRR defective status cannot efficiently repair these double-strand breaks, leading to cell death [6–8]. Another mode of action for PARP inhibitors is to trap PARP proteins at the sites of DNA damage, which is highly toxic to cells due to blockade of DNA replication and induction of a replication stress response. PARP inhibitors proficiently result in synthetic lethality in tumor cells with *BRCA1/2* or other HRR deficiencies, more than in normal DNA repair proficient cells [9, 10]. Rucaparib is one of a series of tricyclic benzimidazole carboxamide PARP inhibitors with a Ki of 1.4 nM for PARP1 in a cell-free assay. It is a poly(ADP-ribose) polymerase (PARP) inhibitor successfully granted a license by the FDA and indicated as a monotherapy for the treatment of patients with a deleterious *BRCA* mutation (germline and/or somatic) associated advanced ovarian cancer who have been treated with two or more chemotherapies [11].

Reactivation of wild-type p53 by preventing the protein-protein binding interaction between p53 and its negative regulator MDM2 induces the growth inhibitory and/or pro-apoptotic functions of p53, and has been demonstrated to have potential as a therapeutic strategy for non-genotoxic activation of p53. Nutlin-3 provided the mechanistic proof-of-concept for small molecule inhibition of the MDM2-p53 interaction and continues to be a useful reference tool compound; however, its potency and pharmacological properties are suboptimal for clinical use [12, 13]. RG7388, a second generation MDM2 inhibitor, was subsequently developed with superior potency, selectivity and oral bioavailability suitable for clinical development, with a cell-free IC_50_ value of 6 nM [14]. These compounds target a small hydrophobic pocket on MDM2, to which p53 normally binds, leading to p53 stabilization and upregulation of p53 downstream transcriptional targets involved in cell cycle arrest and/or apoptosis [15, 16].

Up to 50% to 60% of epithelial ovarian cancer is estimated to be deficient in HRR and hence likely to respond to PARP inhibitors [17]. The approximately 34% of ovarian cancer patients with tumors harboring wild-type *TP53* may benefit from MDM2 inhibitor treatment [16]. Combination chemotherapy for cancer treatment has a long established history, particularly for agents having different mechanism of action and non-overlapping toxicities. Utilizing targeted cancer therapeutic agents in combination is starting to be explored, although it has substantial complexity [18].

In the current study it was hypothesized that combination treatment of Nutlin-3/RG7388 with rucaparib further activates the p53 pathway by inhibition of PARP and results in enhanced induction and stabilization of p53 via Nutlin-3/RG7388 treatment to increase growth arrest and/or apoptosis in wild-type *TP53* ovarian cancer cell lines.

## RESULTS

### The growth inhibitory response of ovarian cancer cell lines to Nutlin-3/RG7388 and rucaparib

**Figure 1:**
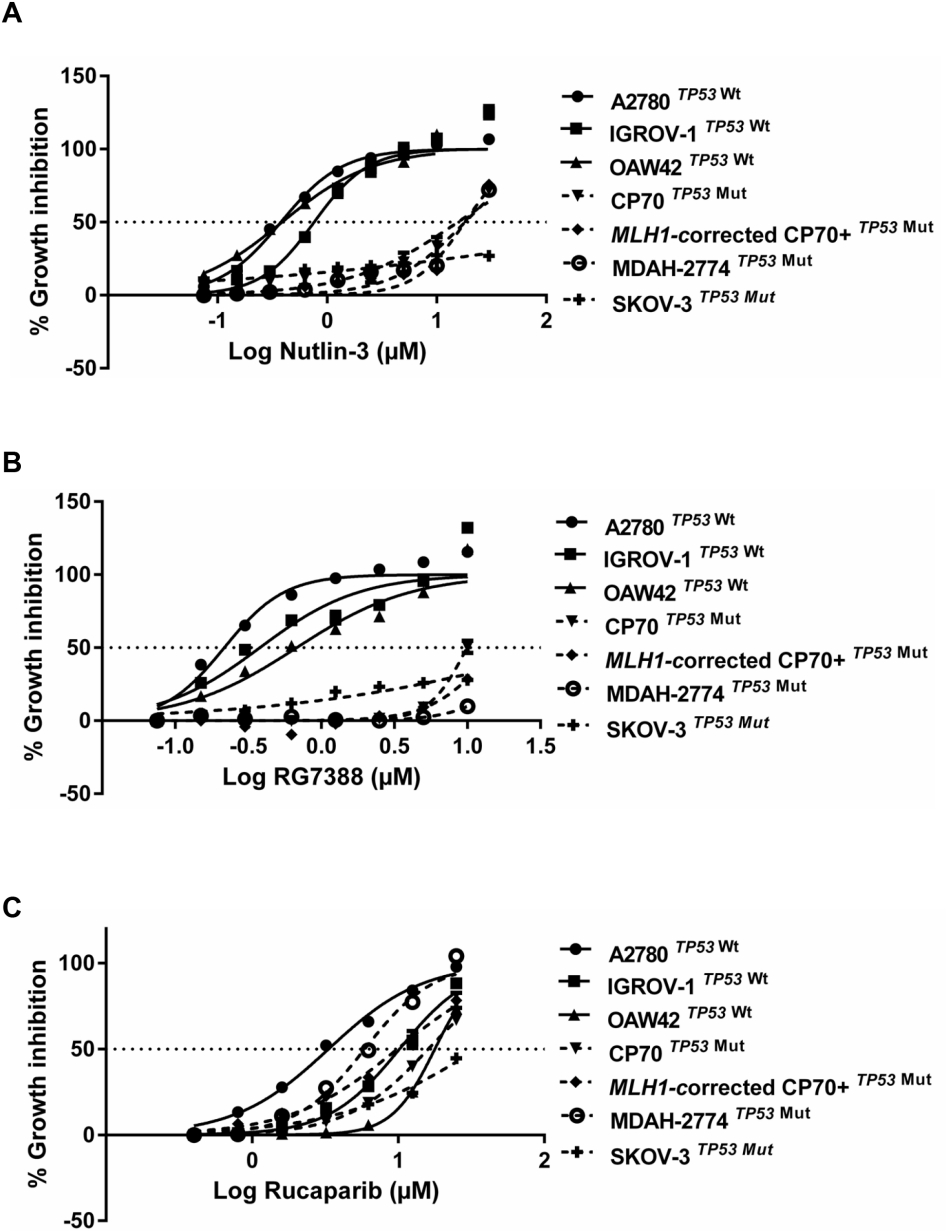
The sensitivity to MDM2 antagonists, Nutlin-3 and RG7388, and rucaparib in a panel of wild-type and mutant *TP53* ovarian cancer cell lines Wild-type *TP53* cell lines are significantly more sensitive to growth inhibition by **(A)** Nutlin-3 (Mann Whitney test, *p< 0.0001*) and **(B)** RG7388 (Mann Whitney test, *p< 0.0001*) treatment for 72 hours compared to mutant *TP53* cell lines. **(C)** The sensitivity to rucaparib is p53-independent. Data shown are the average of at least three independent experiments and error bars represent SEM.

A sulforhodamine-B (SRB) assay was used to investigate growth inhibition by Nutlin-3/RG7388 or rucaparib for a panel of wild-type and mutant *TP53* ovarian cancer cell lines derived from tumors of different histological subtypes [19–22] (Figure 1 and Table 1). The GI_50_ values, required concentration of each compound leading to 50% growth inhibition, showed that wild-type *TP53* ovarian cancer cell lines were significantly more sensitive to Nutlin-3/RG7388 compared to mutant, which is consistent with their mechanism of action (*p<0.0001* Mann-Whitney test) (Figure 1A & 1B). The GI_50_ values for wild-type *TP53* cell lines for RG7388 and Nutlin-3 were in the nanomolar range ( 253.3 ± 73.1 (SEM) nM) and micromolar range (1.76 ± 0.51 (SEM) μM) respectively. In contrast, *TP53* mutant cell lines had GI_50_ values greater than 10 μM (17.8 ± 2.9 (SEM) μM) for RG7388 and range 21.2->30 μM for Nutlin-3 (Table 1).

**Table 1:** GI_50_ concentrations of rucaparib, Nutlin-3 and RG7388 for the panel of ovarian cancer cell lines of varying *TP53* status

Cell line	*TP53* status	Histotype	Rucaparib (μM)	Nutlin-3 (μM)	RG7388 (μM)
**A2780**	Wild-type	Undifferentiated [19]	3.26 ± 0.47	1.23 ± 0.23	0.11 ± 0.01
**IGROV-1**	Wild-type	Mixed, EC with CCC/UD [21]	11.34 ± 0.05	2.8 ± 0.48	0.35 ± 0.04
**OAW42**	Wild-type	Serous Cystadenocarcinoma [20]	19.00 ± 0.58	1.3 ± 0.1	0.31 ± 0.04
**CP70**	Mutant (Heterozygous) c.514 G->T; p.Val172Phe	Undifferentiated [19]	17.00 ± 0.64	21.2 ± 2.5	11.7 ± 1.81
***MLH1*-Corrected CP70+**	Mutant (Heterozygous) c.514 G->T; .Val172Phe	Undifferentiated [19]	14.00 ± 2.84	21.2 ± 1.22	14.5 ± 1.09
**MDAH-2774**	Mutant (Homozygous) c.818G->A; p.Arg273His	Endometrioid carcinoma [22]	6.92 ± 1.48	21.4 ± 0.9	20.7 ± 1.43
**SKOV-3**	Mutant (Homozygous) 265delC; p.Pro89fsX33	Adenocarcinoma [20]	>25	> 30	24.6 ± 1.54

Data represent the mean of at least 3 independent experiments ± SEM. EC, endometrioid carcinoma; CCC, clear cell carcinoma; UD, undifferentiated.

For rucaparib, the cells were treated with a wide range of concentrations (0.4-25 μM) for 72 hours to construct growth inhibition curves and calculate the GI_50_ values. The GI_50_ values significantly varied showing a range of responses, with A2780 (3.26±0.47 μM) and SKOV-3 (> 25 μM) as the most sensitive and resistant cell lines respectively. Although CP70 cells are derived from A2780, they have genetic alterations in addition to the difference in *TP53* genomic status. In particular, CP70 cells are mismatch repair (MMR)-deficient due to loss of the *MLH1* gene and they are resistant to rucaparib even though A2780 cells are MMR-competent and sensitive to rucaparib. The CP70+ cell line is *MLH1*-corrected by chromosome 3 transfer into the CP70 cells and retains the heterozygous *TP53* mutation. The A2780 and derived cell lines differ in growth rate, *TP53* status and *MLH1* status and show differences in response to cisplatin and rucaparib, they are nevertheless all derived from the same tumor and thus overall are genetically closely related. The results of this study showed no relationship between the status of *TP53* and response to rucaparib (Mann-Whitney, p>0.05) (Figure 1C).

### Nutlin-3/RG7388 synergizes with rucaparib for growth inhibition of wild-type *TP53* ovarian cancer cell lines

**Figure 2:**
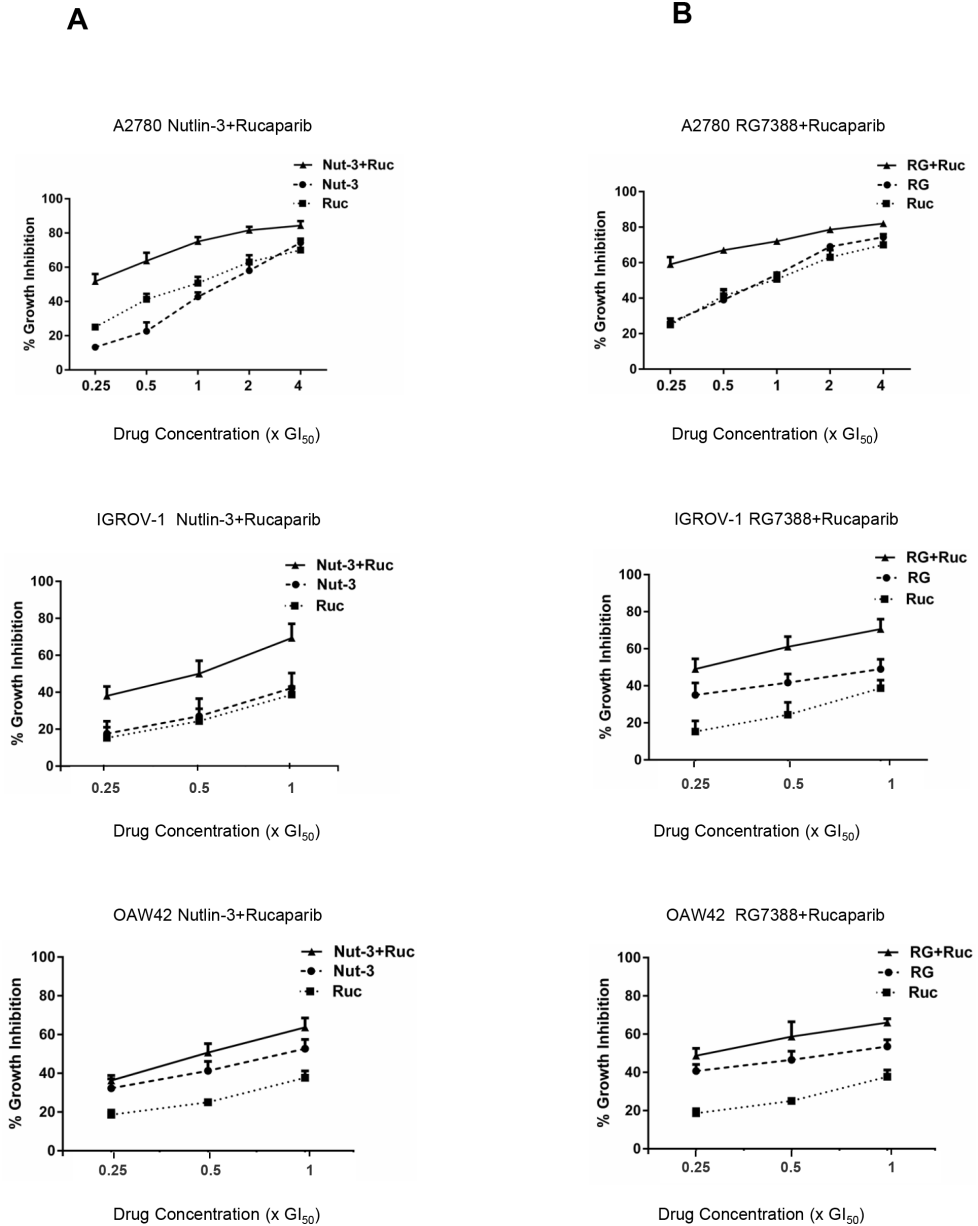
Nutlin-3/RG7388 synergizes with rucaparib in wild-type *TP53* ovarian cancer cells **(A)** Growth inhibition curves of three wild-type *TP53* cell lines exposed to Nutlin-3 and rucaparib alone, and in combination at constant 1:1 ratios of 0.25X, 0.5X, 1X, 2X and 4X (A2780) or 0.25X, 0.5X and 1X (IGROV-1 & OAW42) their respective GI_50_ concentration for 72 hours. **(B)** Growth inhibition curves of three wild-type *TP53* cell lines exposed to RG7388 and rucaparib alone, and in combination at constant 1:1 ratios of 0.25X, 0.5X, 1X, 2X and 4X (A2780) or 0.25X, 0.5X and 1X (IGROV-1 & OAW42) their respective GI_50_ concentrations for 72 hours. Nut-3, Nutlin-3; RG, RG7388; Ruc, rucaparib.

The effect of rucaparib in combination with Nutlin-3/RG7388 was investigated for three wild-type *TP53* ovarian cancer cell lines using median-effect analysis. The sensitivity of the wild-type *TP53* cell lines to growth inhibition during 72 hours exposure to rucaparib and Nutlin-3/RG7388 was determined as single agents, and in combination at 5 equipotent concentrations between 0.25× and 4× their respective GI_50_ concentrations for the A2780 cell line. Owing to the high GI_50_ for IGROV-1 and OAW42 in response to rucaparib, 3 equipotent concentrations between 0.25× and 1x their respective GI_50_ concentrations were used to evaluate the combination effect of rucaparib with Nutlin-3/RG7388. The effect of combined treatment was cell type dependent. The combination of Nutlin-3/RG7388 with rucaparib at all concentrations led to greater growth inhibition compared to either agent alone for the A2780, IGROV-1 and OAW42 cell lines, although the increase was smaller for the OAW42 cells (Figure 2). Combination treatment of rucaparib with Nutlin-3/RG7388 at concentrations equal and lower than the individual 1x GI_50_ dose resulted in more growth arrest compared to doses higher than 1x GI_50_ for the A2780 cell line.

**Figure 3:**
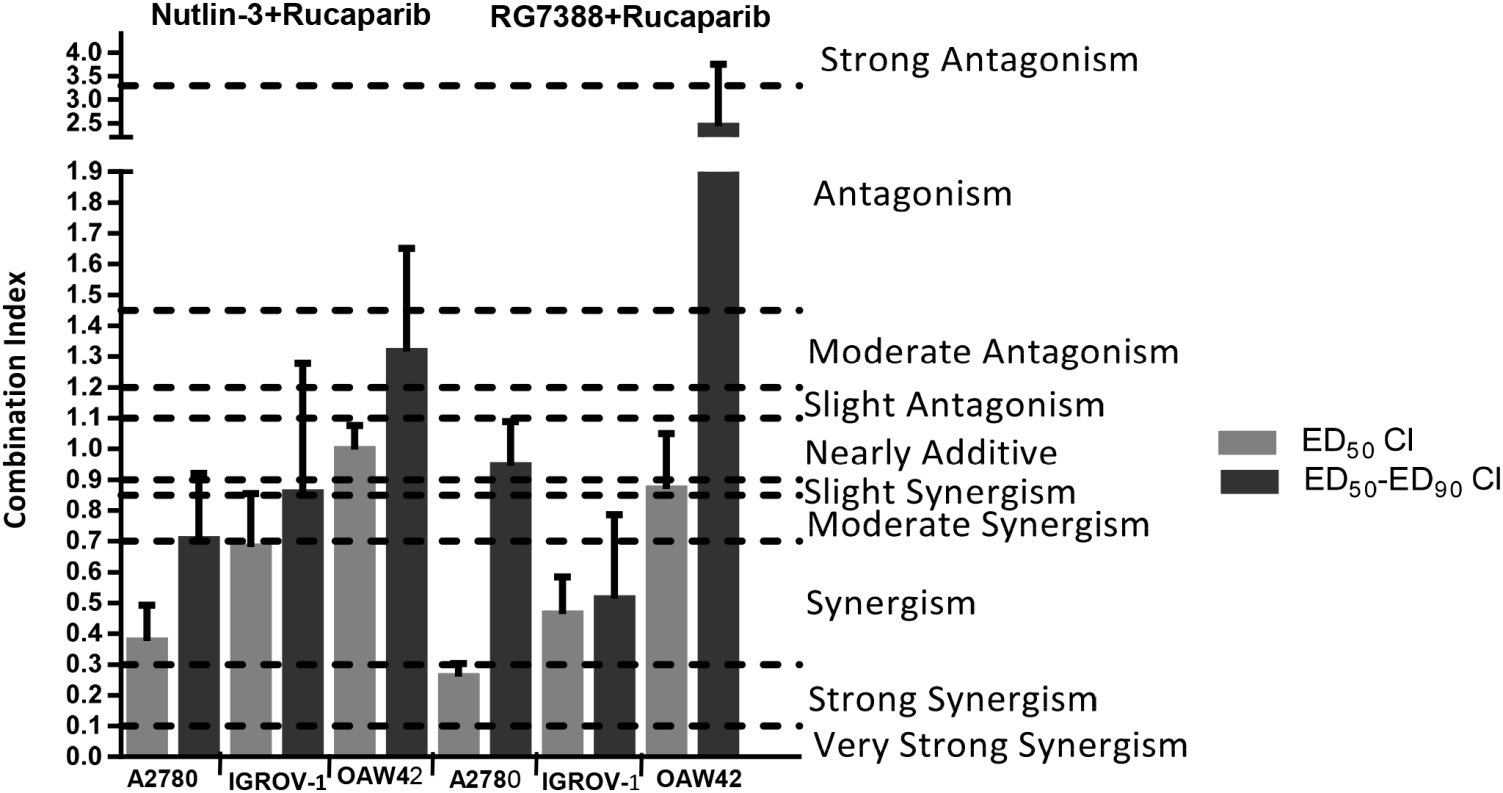
The CI values for Nutlin-3/RG7388 in combination with rucaparib at ED_50_ and the average of CI values at effect levels ED_50_, ED_75_ and ED_90_ in three wild-type TP53 ovarian cancer cell lines Data are shown as the average of at least 3 independent experiments and error bars represent SEM. CI, Combination Index; ED, Effective Dose.

To determine whether the observed differences in growth inhibition were additive, synergistic or antagonistic, the data were analyzed using median-effect analysis and Combination Index (CI) values calculated. CI values for each constant ratio combination and at effect levels of ED_50_, ED_75_ and ED_90_ were computed and the average of CI values at ED_50_, ED_75_ and ED_90_ was also determined (Figure 3 & Table 2). Combined treatment of rucaparib with Nutlin-3/RG7388 ranged from additive to strong synergism based on the CI at ED_50_ and overall CI for A2780 and IGROV-1 cell lines, whereas only slight synergism to antagonism was observed for the OAW42 cell line (Figure 3 & Table 2). Interestingly, rucaparib, Nutlin-3 and RG7388 had favorable Dose Reduction Index (DRI) values for combined treatment with all experimental values ranging from 1.2-fold to 7.8-fold dose reduction (Table 3).

**Table 2:** Growth inhibition CI values for RG7388/Nutlin-3 in combination with rucaparib for the wild-type *TP53* ovarian cancer cell lines

Cell Line	Combination	CI					CI ED_50_	CI ED_75_	CI ED_90_	CI Ave ED_50-90_
		XGI_50_								
		0.25	0.5	1	2	4				
**A2780**	Nutlin-3+Rucaparib	**0.4**	**0.5**	**0.5**	**0.6**	1.0	**0.4**	**0.6**	1.1	**0.7**
	RG7388+Rucaparib	**0.4**	**0.4**	**0.6**	**0.7**	1.2	**0.3**	**0.7**	1.9	**0.9**
**IGROV-1**	Nutlin-3+Rucaparib	**0.6**	**0.7**	**0.6**	ND	ND	**0.7**	**0.8**	1.1	**0.9**
	RG7388+Rucaparib	**0.4**	**0.4**	**0.3**	ND	ND	**0.5**	**0.4**	**0.7**	**0.5**
**OAW42**	Nutlin-3+Rucaparib	**0.9**	**0.7**	**0.9**	ND	ND	1.0	1.3	1.7	1.3
	RG7388+Rucaparib	**0.9**	1.0	1.4	ND	ND	1.2	3.2	4.6	2.4

The combined treatment was performed at the indicated fixed 1:1 ratios relative to their respective GI_50_ concentrations. CI values were calculated for each constant ratio combination and at effect levels ED_50_, ED_75_ and ED_90_ from the average of at least three independent experiments. CI Ave ED_50-90_ represents the average of CI values at effect levels of ED_50_, ED_75_ and ED_90_. CI range: < 0.1 very strong synergism; 0.1-0.3 strong synergism; 0.3-0.7 synergism; 0.7-0.85 moderate synergism; 0.85-0.9 slight synergism; 0.9-1.1 nearly additive; 1.1-1.2 slight antagonism; 1.2-1.45 moderate antagonism; 1.45-3.3 antagonism; 3.3-10 strong antagonism; > 10 very strong antagonism. Synergistic combinations are highlighted in bold font. CI, Combination Index; ED, Effective Dose.

**Table 3:** DRI values for growth inhibition by RG7388/Nutlin-3 in combination with rucaparib for the wild-type *TP53* ovarian cancer cell lines

Cell Line	Combination	Component	DRI				
			XGI_50_				
			0.25	0.5	1	2	4
**A2780**	Nutlin-3+Rucaparib	Rucaparib	**3.1**	**2.7**	**3.0**	**2.5**	**1.6**
		Nutlin-3	**5.00**	**3.9**	**3.6**	**2.6**	**1.6**
	RG7388+Rucaparib	Rucaparib	**5.4**	**4.0**	**3.1**	**2.5**	**1.4**
		RG7388	**5.1**	**4.4**	**3.0**	**2.4**	**1.6**
**IGROV-1**	Nutlin-3+Rucaparib	Rucaparib	**3.5**	**3.0**	**3.9**	ND	ND
		Nutlin-3	**3.5**	**2.8**	**3.3**	ND	ND
	RG7388+Rucaparib	Rucaparib	**5.7**	**5.0**	**3.7**	ND	ND
		RG7388	**2.7**	**3.9**	**7.8**	ND	ND
**OAW42**	Nutlin-3+Rucaparib	Rucaparib	**2.3**	**1.6**	**1.9**	ND	ND
		Nutlin-3	**1.9**	**1.2**	**1.2**	ND	ND
	RG7388+Rucaparib	Rucaparib	**4.3**	**4.0**	**3.0**	ND	ND
		RG7388	**2.0**	**2.1**	**1.8**	ND	ND

The combined treatment was performed at the indicated fixed 1:1 ratios relative to their respective GI_50_ concentrations. DRI values were calculated for each constant ratio combination from the average of at least three independent experiments. Favorable DRI values (>1.0) are highlighted in bold font. DRI, Dose Reduction Index.

### The effect of combination treatment with rucaparib and Nutlin-3/RG7388 on activation of the p53 pathway

**Figure 4:**
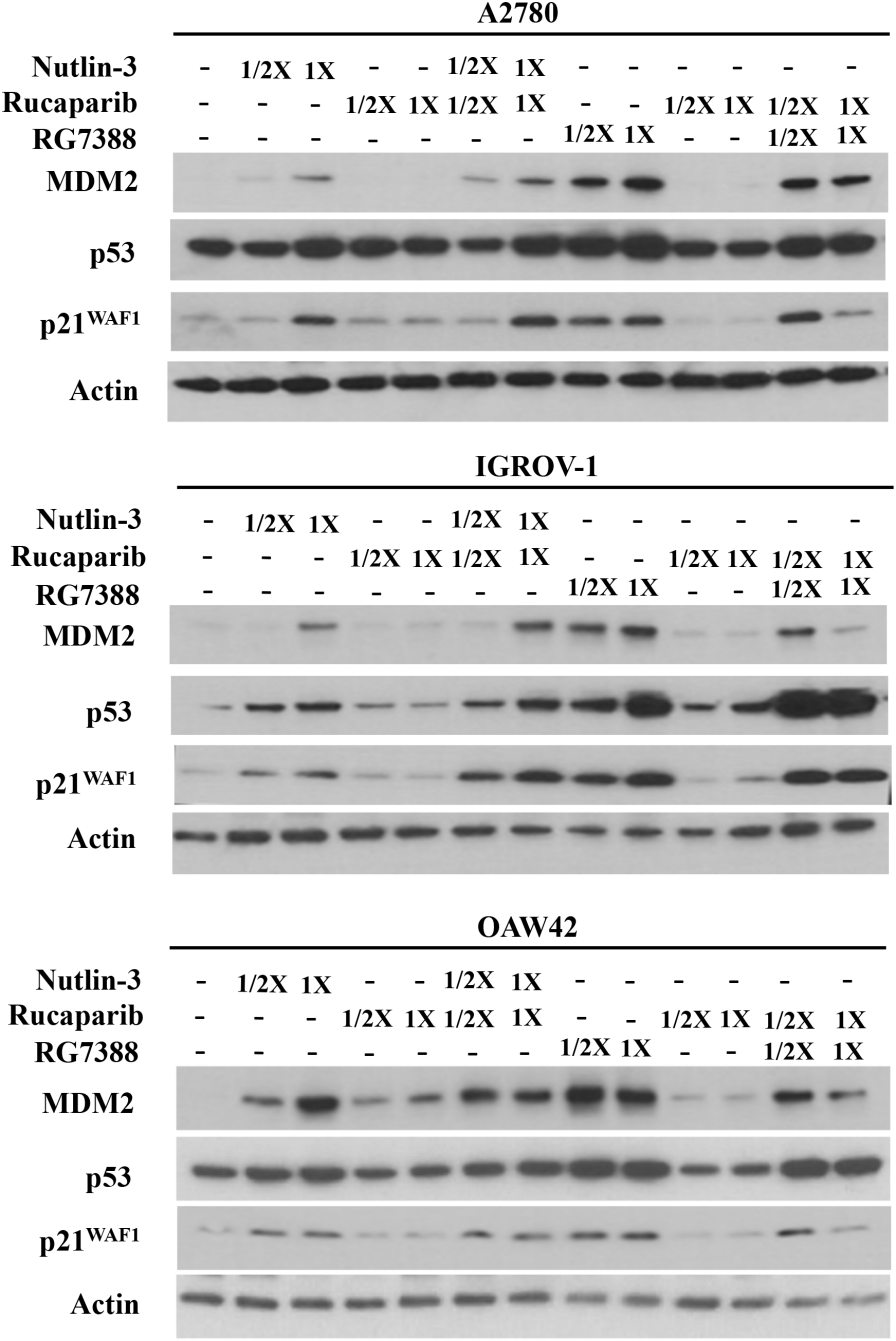
Combination of Nutlin-3/RG7388 with rucaparib increased stabilization of p53 and upregulation of its downstream targets, MDM2 and p21^WAF1^ compared to rucaparib on its own but not compared to Nutlin-3/RG7388 Total levels of p53, p21^WAF1^, MDM2 4 hours after the commencement of treatment with Nutlin-3 and RG7388 alone, and in combination with rucaparib at constant 1:1 ratios of 1/2X and 1X their respective GI_50_ concentration analyzed by western blot in three wild-type *TP53* ovarian cancer cell lines.

**Figure 5:**
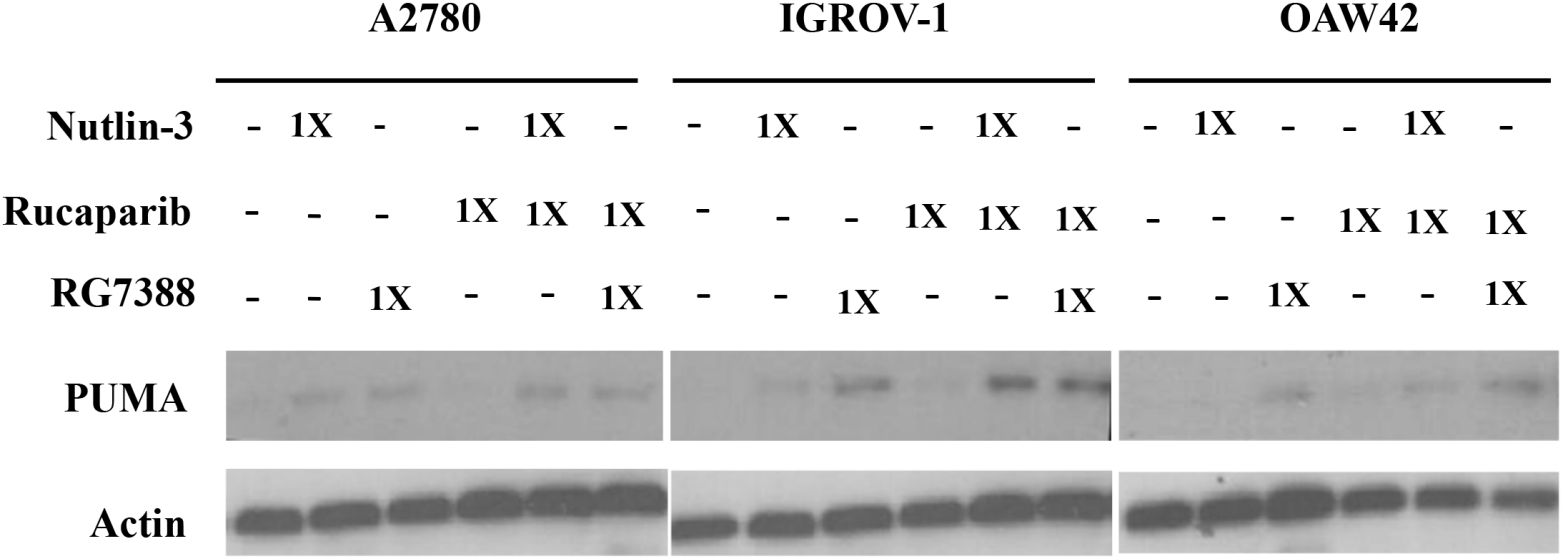
Combination of Nutlin-3/RG7388 with rucaparib increased upregulation of *TP53* downstream target, PUMA compared to rucaparib on its own but not compared to Nutlin-3/RG7388 Total levels of PUMA 24 hours after the commencement of treatment with Nutlin-3 and RG7388 alone, and in combination with rucaparib at constant 1:1 ratios of 1X their respective GI_50_ concentration analyzed by western blot in three wild-type *TP53* ovarian cancer cell lines.

Western blotting was used to investigate the effect of combination treatment on the p53 molecular pathway. Wild-type *TP53* cell lines were treated with rucaparib, Nutlin-3 or RG7388 alone, and in combination at constant 1:1 ratios of 1/2x and 1x their respective GI_50_ concentrations for 4 hours. They were also treated with rucaparib, Nutlin-3 or RG7388 alone, and in combination at constant 1:1 ratios of 1x their respective GI_50_ concentrations for 24 hours, to test the induction of PUMA (BBC3) as a *TP53*-related pro-apoptotic gene. Western blot analysis showed that rucaparib treatment as a single agent had no effect on p53 stabilization, upregulation of p21^WAF1^, MDM2 or PUMA compared to DMSO control, nor generally did rucaparib increase the effect of the MDM2 inhibitors on the p53 pathway (Figure 4 & Figure 5). Although rucaparib in combination with Nutlin-3/RG7388 appeared to increase stabilization of p53 and its downstream transcriptional targets in some cases in IGROV-1, otherwise there were no convincing differences. No evidence of synergy was observed at the molecular level to indicate that the mechanism of synergy for growth inhibition and apoptosis by rucaparib involved enhancement of the p53 pathway activation by MDM2 inhibitors (Figure 4 & Figure 5).

### Rucaparib in combination with Nutlin-3/RG7388 induces cell cycle distribution changes and/or apoptosis in wild-type *TP53* ovarian cancer cell lines

Wild-type *TP53* cell lines were treated with rucaparib and Nutlin-3/RG7388, alone and in simultaneous combination at constant 1:1 ratios of 1/2x and 1x their respective GI_50_ concentrations for 24, 48 and 72 hours. Then, they were analyzed by flow cytometry for cell cycle phase distribution changes and evidence of apoptosis in response to treatment.

#### Combination with Nutlin-3

**Figure 6:**
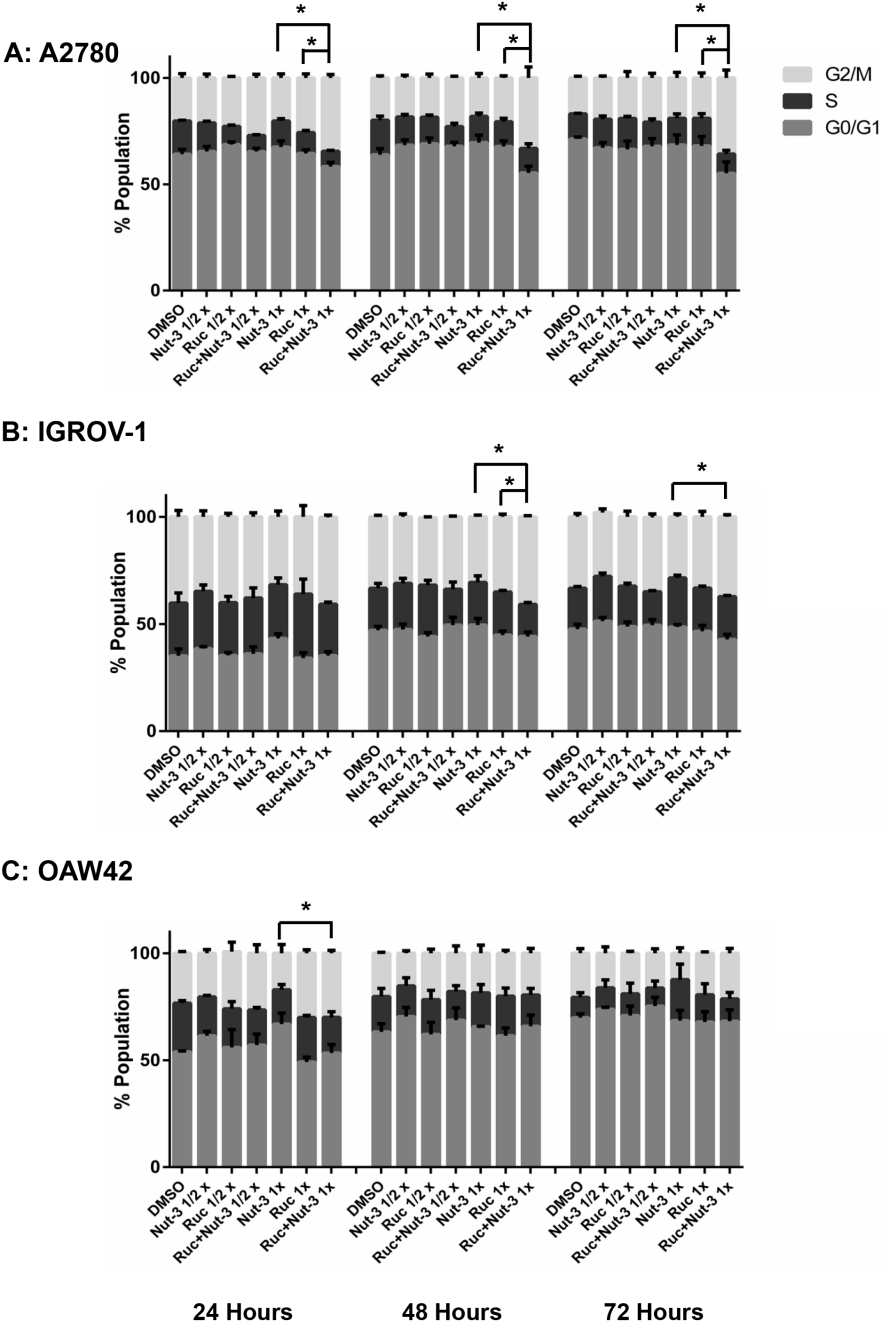
Combination of Nutlin-3 with rucaparib affects the cell cycle distribution Wild-type *TP53* ovarian cancer cells were treated for 24, 48 and 72 hours with Nutlin-3 or rucaparib alone and at constant 1:1 combination ratios of 1/2 X & 1X their respective GI_50_ concentrations. **(A)** A2780 cell line, **(B)** IGROV-1 cell line, **(C)** OAW42 cell line. Nut-3, Nutlin-3; Ruc, rucaparib; *, *p* < *0.05*. Data are shown as the average of at least 3 independent experiments and error bars represent SEM.

Rucaparib on its own slightly increased the proportion of cells in G2/M phase and the number of SubG1 events in a dose and time-dependent manner. Combination treatment of rucaparib with Nutlin-3 resulted in an increased percentage of cells in the G2/M cell cycle phase compared to either agent alone, in a treatment time and dose-dependent manner for A2780 and IGROV-1 cell lines (Figure 6A & 6B). For the OAW42 cell line after 24 hours, the combination treatment led to an increased proportion of the cell population in the G2/M phase of the cell cycle compared to Nutlin-3 as a single agent. After 48 and 72 hours, there was little change in the cell cycle distribution following combination of rucaparib with Nutlin-3 compared to either agent alone (Figure 6C).

**Figure 7:**
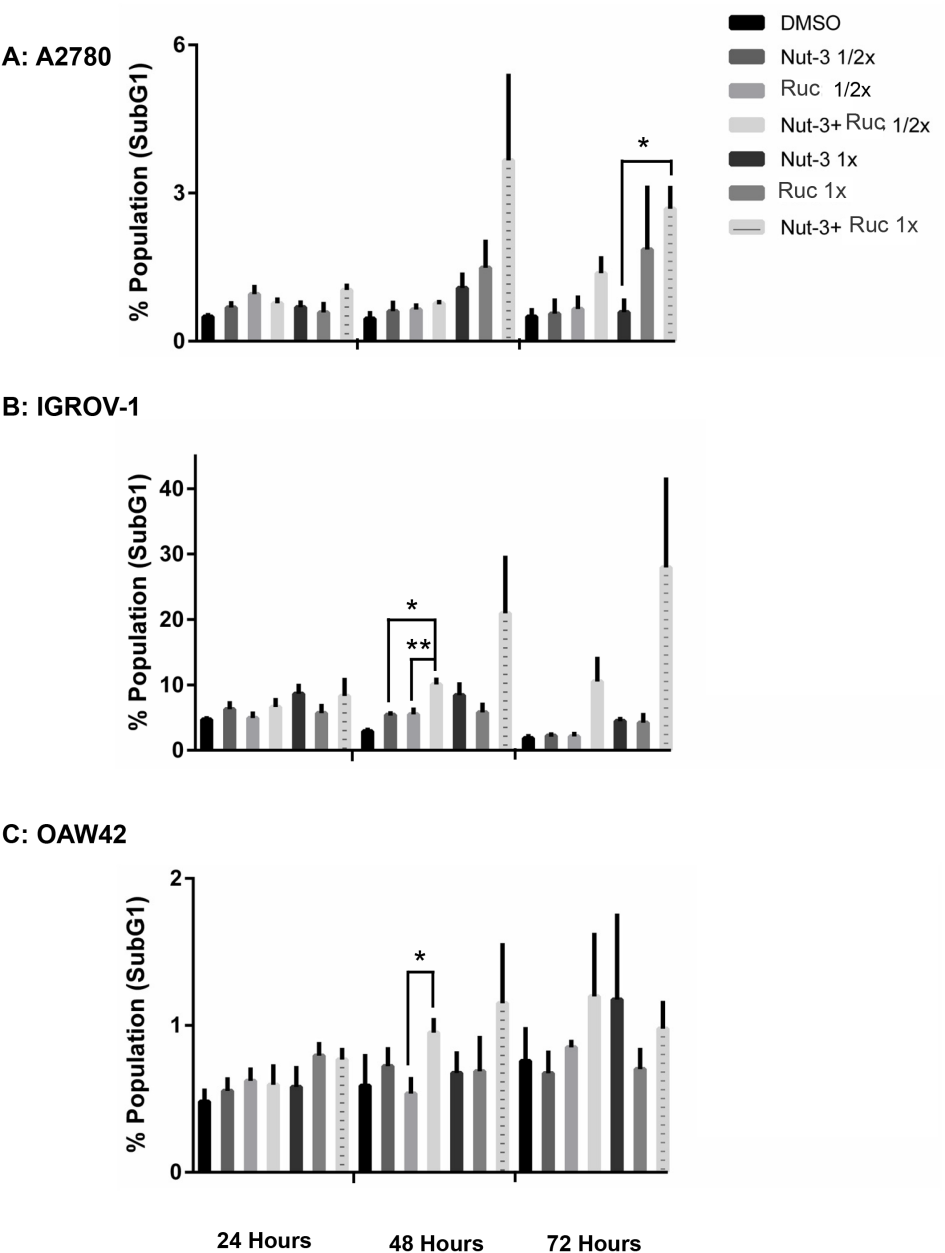
Combination of Nutlin-3 with rucaparib affects the apoptotic endpoints FACS analysis for SubG1 events. Wild-type *TP53* ovarian cancer cells were treated for 24, 48 and 72 hours with Nutlin-3 or rucaparib alone and at constant 1:1 combination ratios of 1/2 X & 1X their respective GI_50_ concentrations. **(A)** A2780 cell line, **(B)** IGROV-1 cell line, **(C)** OAW42 cell line. Nut-3, Nutlin-3; Ruc, rucaparib; *, *p* <*0.05*; **, *P < 0.01*. Data are shown as the average of at least 3 independent experiments and error bars represent SEM.

The basal levels of apoptosis, indicated by SubG1 signals on FACS analysis, differed markedly between the cell lines, with much higher basal levels evident with the IGROV1 cells and least for the OAW42 cells. However, across all 3 cell lines, the trend was for combination treatments to induce an increased number of SubG1 signals in a concentration and time-dependent manner (Figure 7).

#### Combination with RG7388

**Figure 8:**
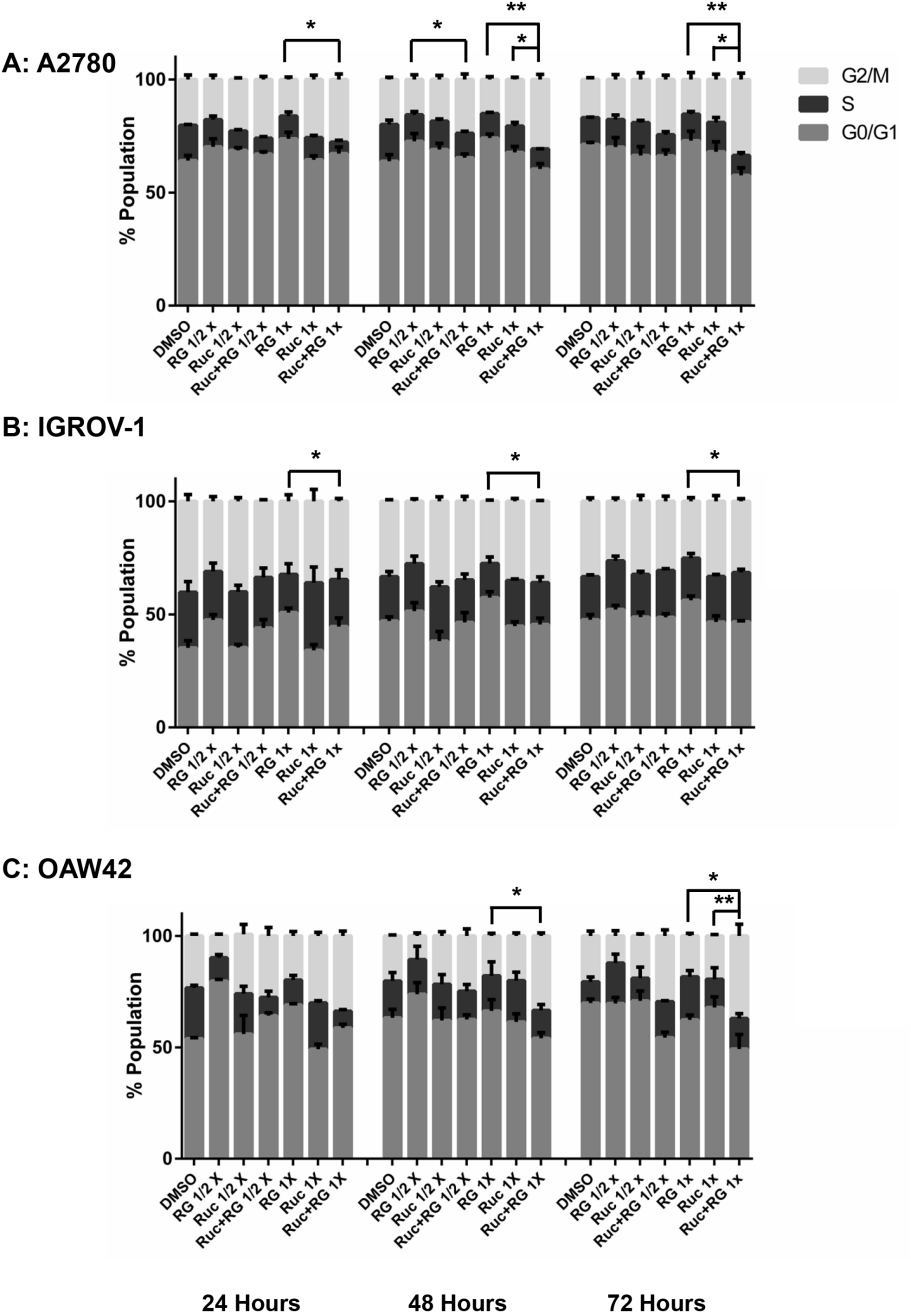
Combination of RG7388 with rucaparib affects the cell cycle distribution Wild-type *TP53* ovarian cancer cells were treated for 24, 48 and 72 hours with RG7388 or rucaparib alone and at constant 1:1 combination ratios of 1/2 X & 1X their respective GI_50_ concentrations. **(A)** A2780 cell line, **(B)** IGROV-1 cell line, **(C)** OAW42 cell line. RG, RG7388; Ruc, rucaparib; *, *p* <*0.05*; **, *P < 0.01*. Data are shown as the average of at least 3 independent experiments and error bars represent SEM.

**Figure 9:**
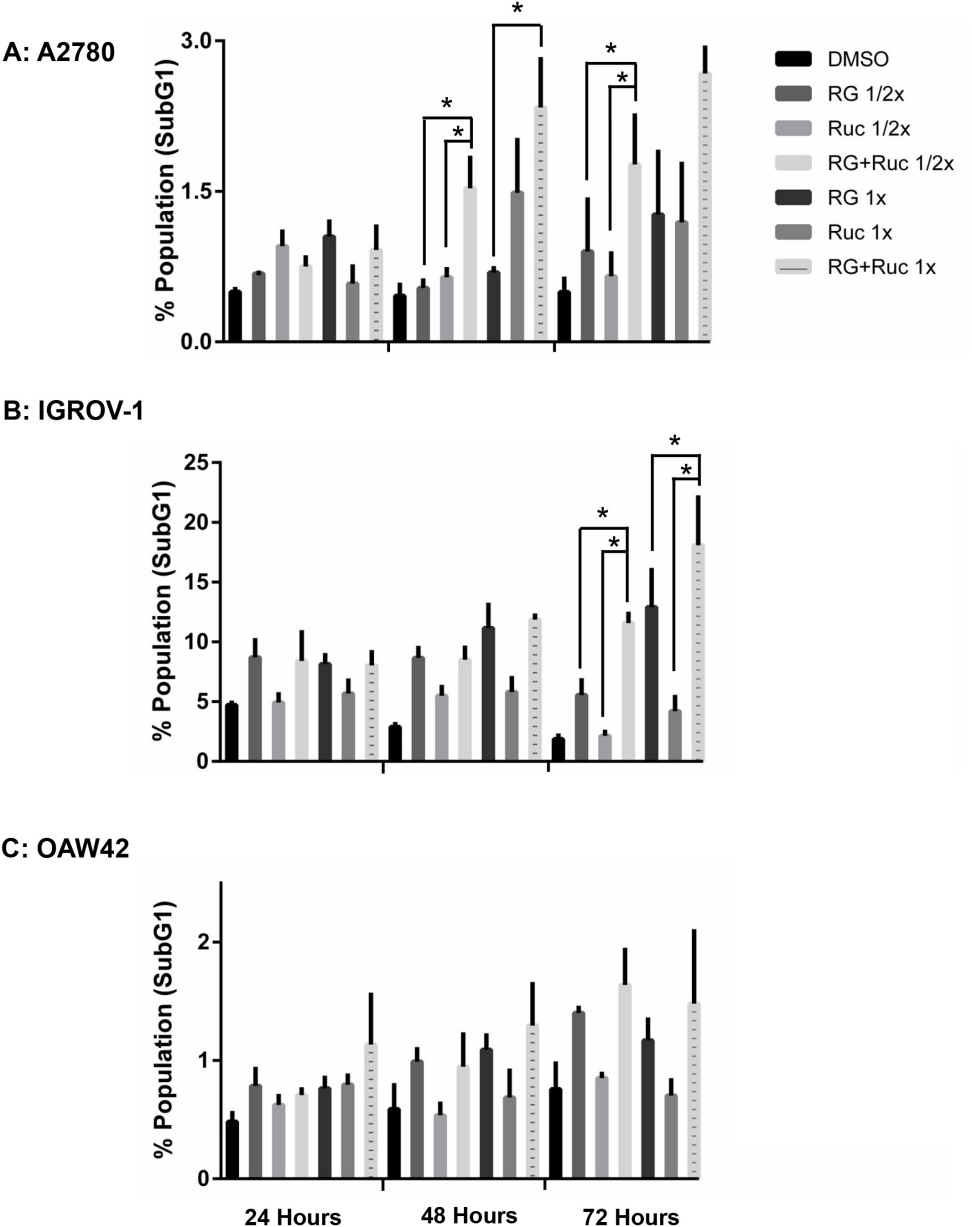
Combination of RG7388 with rucaparib affects the apoptotic endpoints FACS analysis for SubG1 events. Wild-type *TP53* ovarian cancer cells were treated for 24, 48 and 72 hours with RG7388 or rucaparib alone and at constant 1:1 combination ratios of 1/2 X & 1X their respective GI_50_ concentrations. **(A)** A2780 cell line, **(B)** IGROV-1 cell line, **(C)** OAW42 cell line. RG, RG7388; Ruc, rucaparib; *, *p* <*0.05*. Data are shown as the average of at least 3 independent experiments and error bars represent SEM.

For A2780 cells, the combination of rucaparib with RG7388 increased the proportion of cells in G2/M phase and SubG1 signals compared to either agent alone, in a treatment time and dose-dependent manner. The combination treatment also decreased the proportion of cells in S-phase compared to either agent alone (Figure 8A & 9A). In terms of the proportional distribution of IGROV-1 cells in G0/G1 or G2/M, the effect of rucaparib combination with RG7388 was time dependent. Combined treatment for 24 and 48 hours led to proportionally more cells in G0/G1 compared to the effect of rucaparib on its own and a higher proportion of cells in G2/M compared to the effect of RG7388 alone. After 72 hours treatment, the combination of rucaparib with RG7388 resulted in increased G2/M cell cycle arrest compared to RG7388 on its own, with little change in the percentage of cells in G0/G1 cell cycle compared to rucaparib alone. Combined treatments also increased the percentage of SubG1 signals compared to either agent alone, in a time and dose-dependent manner (Figure 8B & 9B). For OAW42 cells, combination of rucaparib with RG7388 led to proportionally more G2/M cells compared to either agent alone. It also decreased the percentage of cells in S-phase compared to either agent alone after 24 and 48 hours (Figure 8C & 9C).

**Figure 10:**
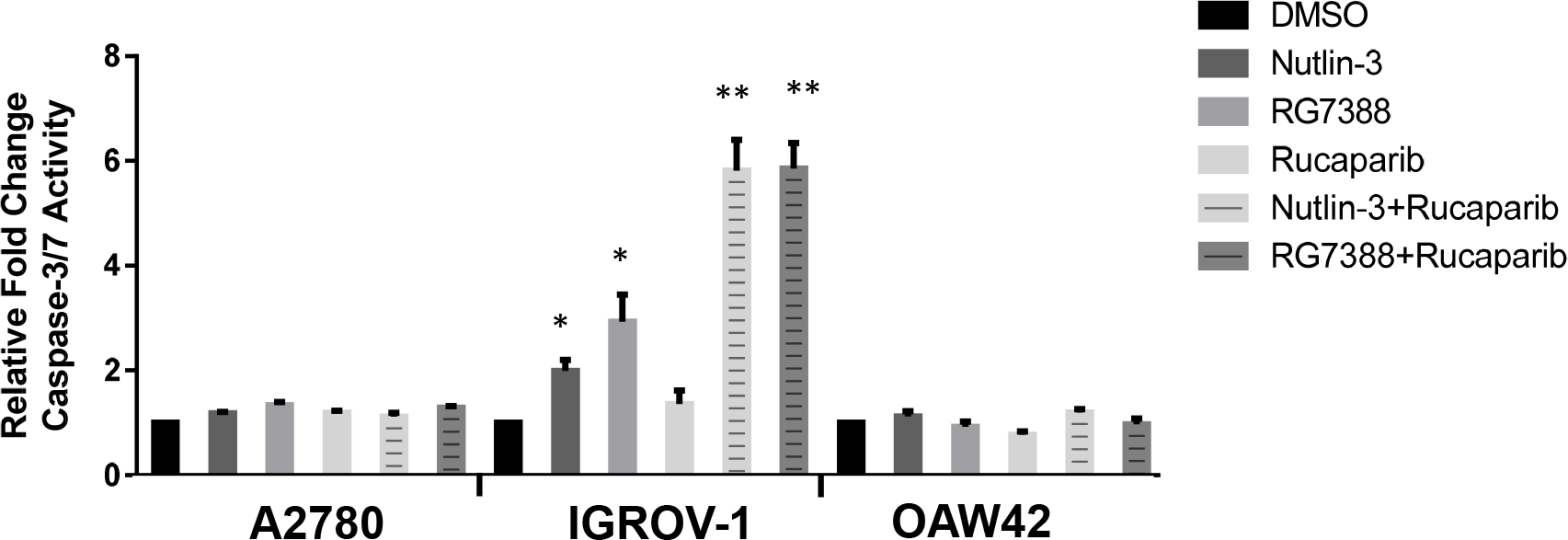
Combinations of Nutlin-3 or RG7388 with rucaparib affect caspase3/7 activity The wild-type *TP53* ovarian cancer cells treated at constant 1:1 ratios of 1x their respective GI_50_ concentrations of Nutlin-3, RG7388 or rucaparib alone, and in combination for 24 hours. Caspase 3/7 activity is represented as fold change relative to DMSO solvent control. *, The single star represents a significant increase in the caspase3/7 activity compared to DMSO control (*p<0.05*); **, The double stars represent a significant increase in the caspase3/7 activity compared to Nutlin-3, RG7388 or rucaparib alone (*p<0.01*).

Caspase 3/7 enzymatic activity was assessed to evaluate induction of apoptosis. Wild-type *TP53* ovarian cancer cell lines were treated for 24 hours with 1x their respective Nutlin-3/RG7388 GI_50_ concentrations as a single agent and in combination with rucaparib (Figure 10). For A2780 and OAW42 there was no significant increase in the caspase 3/7 activity in response to Nutlin-3/RG7388 alone. Also no significant increase was observed for the combination of Nutlin-3/RG7388 with rucaparib compared with the effect of either agent alone in the A2780 and OAW42 cells. In contrast, there was a significant increase in caspase 3/7 activity in response to both Nutlin-3 and RG7388 as single agents in IGROV-1. Furthermore, the combination of Nutlin-3/RG7388 with rucaparib resulted in more caspase 3/7 activity in IGROV-1 compared to either agent alone (Figure 10).

## DISCUSSION

PARP inhibitors have been developed over a number of decades and in more recent years have been shown to have efficacy as single agents against tumor cells with intrinsic deficiencies in DNA repair. They currently have been undergoing clinical trials in different types of cancers, including ovarian cancer [23–25]. Encouraging clinical trial results for the use of PARP inhibitors have been reported for ovarian cancer [11, 26]. The promising results showing activity of MDM2-p53 antagonists against wild-type *TP53* ovarian cancer cell lines [16, 27] have prompted this investigation of combination treatment with rucaparib and Nutlin-3/RG7388 in a panel of wild-type *TP53* ovarian cancer cell lines. Mechanistically it was of interest to investigate the combined effect of PARP inhibition and MDM2-p53 binding antagonists on the p53 pathway activation. This study evaluates for the first time the effect of the MDM2-p53 binding antagonists Nutlin-3 and RG7388 in combination with rucaparib in wild-type *TP53* ovarian cancer cell lines and explores the interplay between the p53 pathway and PARP dependent pathways modulated by rucaparib.

Within the panel of ovarian cancer cell lines studied, wild-type *TP53* cell lines were significantly more sensitive to Nutlin-3 and the more potent RG7388 compared to mutant *TP53* cell lines, which is consistent with their mechanism of action [28]. Following the scheme suggested by Mukhopadhyay et al, the 10 μM cut-off value was used to categorize cell lines into sensitive and resistant to PARP inhibitor [17]. Among the individual cell lines, A2780 and MDAH-2774 were sensitive (GI_50_ < 10 μM) and other cell lines (IGROV-1, OAW42, CP70, *MLH1*-corrected CP70+ and SKOV-3) were resistant (GI_50_ > 10 μM). No relationship was found between the *TP53* status of cells and response to rucaparib. These results are in line with those of previous studies reporting the sensitivity of A2780 and resistance of OAW42, IGROV-1, CP70 and SKOV-3 in response to rucaparib [29, 30]. Overall, the GI_50_ values for rucaparib across all cell lines were much higher than the concentration needed to reduce the cell-free enzymatic activity by half, Ki=1.4 nM [31]. However, they were in the range of rucaparib concentrations achievable *in vivo* and used in clinical trials [10, 11, 26, 32].

To explore the potential mechanism involved in the sensitivity of A2780 and MDAH-2774 and resistance of other cell lines to rucaparib, the status of genes implicated as biomarkers in response to PARP inhibitors were studied [29, 33–37]. The most interesting finding is that the A2780 cell line was the most sensitive cell line even though among the relevant genes previously implicated in the sensitivity to rucaparib it has only a heterozygous mutation in *PTEN* (Deletion-In frame, heterozygous, (c.380_388delGAAAGGGAC) (89682879_89682887delAGGGACGAA) (Supplementary Table 1). MDAH-2774 is an ovarian endometrioid tumor with mutant *TP53* and *KRAS* [22].

Overall, the reasons for the greater sensitivity of A2780 and MDAH-2774 to rucaparib are not clear. Further research is needed to identify reliable biomarkers to stratify patients who will benefit from treatment with PARP inhibitors.

Resistance to MDM2-p53 binding antagonists has been suggested to be acquired by prolonged exposure of cells to sub-lethal doses through *de novo* inactivating *TP53* mutations or selection of pre-existing subclones of *TP53* mutant cells that might be present as a result of cancer cell genomic instability and tumor heterogeneity [38, 39]. Development of resistance to PARP inhibitors occurs through different mechanisms. For example, acquired resistance to PARP inhibitors resulting from a secondary mutation of the *BRCA* gene has been confirmed to occur in patients [10]. Combination therapy is suggested to delay or prevent drug resistance. Other major benefits of combination therapy are the potential for a synergistic therapeutic effect, or at least the possibility of dose and toxicity reduction [40, 41].

This study set out with the aim of assessing the effect of combination treatment of Nutlin-3 or RG7388 with rucaparib in a panel of ovarian cancer cell lines of known *TP53* status as combined targeted therapeutics. Updated results from the ARIEL2 clinical trial of rucaparib in 152 patients with wild-type *BRCA*1/2 who were sensitive to platinum reported a response rate of 36% in those with BRCA-like DNA repair deficiency status. Less common mutations involved in Homologous Recombination Deficiency (HRD) and predictive of response to platinum may be present in almost one third (33%) of ovarian cancer patients [42]. Almost 30% of ovarian cancers include histological subtypes other than high grade serous and these are mostly *TP53* wild-type. Thus nearly 10% of ovarian cancer patients (one third of the 30% not high grade serous) who are likely to be sensitive to both MDM2 inhibitors and rucaparib may benefit from combination treatment with these agents. In addition, almost 4% of high grade serous ovarian cancers nevertheless have wild-type *TP53* and these mostly have *BRCA1/2* mutation or other HRD status [42], rendering them sensitive to both MDM2 inhibitors and rucaparib and therefore likely to benefit from the combined treatment. Furthermore, those ovarian tumours which include a histological mixture of high grade serous with clear cell, mucinous, endometrioid or low grade serous might gain benefit from this combination treatment to target the different components.

Overall, the effect of combined treatment was cell type and exposure time dependent, with a higher synergistic effect for the combination of Nutlin-3/RG7388 with rucaparib for A2780 and IGROV-1 cell lines. Interestingly, although both IGROV-1 and OAW42 were resistant to rucaparib, there was nevertheless a synergistic effect for the combination of Nutlin-3/RG7388 with rucaparib. A possible explanation for this is that the defects conferring sensitivity to PARP inhibitors as single agents may be different from those which play an important role in the response to the combination treatment. For example, serious deficiencies in HRR may affect the sensitivity to PARP inhibitors as single agents, while mild defects in HRR may have no effect on response to PARP inhibitors alone but may nevertheless influence the effect of combination treatments [8, 29]. Another possibility is that different p53-dependent off-target effects of these MDM2 inhibitors [43, 44] and/or off-target effects of rucaparib with respect to PARP1 and PARP2 may influence the growth inhibitory effect of combined treatment compared to rucaparib as a single agent [45, 46].

An important and clinically relevant finding from the data is the favorable DRI values in both combination treatment of Nutlin-3 or RG7388 with rucaparib. Additive and even mildly antagonistic results of combined treatment can nevertheless be of potential clinical use due to favorable DRI values [47, 48]. These favorable DRI values demonstrate that combination of Nutlin-3 or RG7388 with rucaparib has the potential to reduce the dose of drugs in most cases to achieve the same overall level of effect, indicating a potential clinical benefit of combining these therapeutic agents, particularly when they have differing dose limiting toxicities.

Rucaparib on its own had no effect on p53 stabilization and upregulation of its downstream targets p21^WAF1^, MDM2 and PUMA across all three cell lines. Combination treatment of Nutlin-3 or RG7388 with rucaparib induced stabilization of p53 and upregulation of p21^WAF1^, MDM2 and PUMA compared to rucaparib on its own, whereas rucaparib had no enhancement of the p53 activation by MDM2 inhibitors alone. These results demonstrate that the synergistic effect on growth inhibition observed for the combination of rucaparib and Nutlin-3/RG7388 is not the result of an accentuated p53 response and hence not related to the p53 molecular pathway. The interplay between PARP and p53 is controversial which may be related to the type of DNA damage, type of PARP inhibitors and intensity of replicative stress [49, 50].

Individually, Nutlin-3 and RG7388 induced cell cycle arrest in wild-type *TP53* ovarian cancer cell lines in a time and dose-dependent manner. Rucaparib had little effect on the cell cycle distribution for IGROV-1 and OAW42 cell lines, which is in agreement with the results obtained by Porcelli et al. [51] that indicated no effect of rucaparib on the cell cycle progression of pancreatic cancer cells. However, in the current study rucaparib significantly decreased the proportion of cells in S-phase in A2780 cells, consistent with a recent study indicating a robust decrease in the percentage of cells in S-phase following treatment of U2OSDR-GFP cells with olaparib [50]. Furthermore, there was only a slight increase in the SubG1 cell subpopulation across all cell lines treated with rucaparib compared to DMSO control, suggesting that cells are not undergoing apoptosis. These results are in line with those of Jelinic and Levine who observed low SubG1 events in cancer cells treated with olaparib or veliparib [49].

Across all three cell lines, combined treatment of Nutlin-3/RG7388 with rucaparib increased the proportion of cells in the G2/M phase of the cell cycle, which was marked for A2780 and IGROV-1. This can be explained by induction of p21^WAF1^ expression in response to MDM2 inhibitors and its critical role in cell cycle arrest in G2/M by inhibiting mitotic Cdk complexes and Rb phosphorylation [52]. Also, p53-mediated repression of FoxM1 results in the maintenance of a stable G2/M arrest which is partially p21 dependent [53]. The increased proportion of cells in G2/M phase following PARP inhibitor treatments may reflect their mechanism of action during DNA replication in S-phase, which is to trap PARP-1 and -2 to DNA and induce a replicative stress response [50]. Combination treatment with Nutlin-3 or RG7388 and rucaparib also led to more SubG1 events and/or higher levels of caspase 3/7 activity compared to either agent alone in a cell type dependent manner which was marked for IGROV-1 cells.

In conclusion, the present study demonstrates that combination treatment with MDM2 inhibitors and rucaparib has synergistic and/or dose reduction potential dependent on cell genotype and compound and merits further investigation. Monitoring HRR status, cell cycle markers, PARP expression and PARP activity are likely to provide additional useful information to assess the effectiveness of PARP inhibitors. This information may be helpful to stratify the patients who might benefit from PARP inhibitors and their potential combination with the emerging class of MDM2 inhibitors.

## MATERIALS AND METHODS

### Chemicals and antibodies

Nutlin-3, a 1:1 mixture of the active enantiomer Nutlin-3a and the inactive enantiomer Nutlin-3b, was purchased from NewChem (Newcastle, UK) and RG7388 (Idasanutlin) was synthesized by Ruth Bawn and Amy Heptinstall within the Newcastle Drug Discovery Group. Both were dissolved in dimethyl sulfoxide (DMSO). Rucaparib was kindly supplied by Clovis Oncology, and prepared in 10mM stocks solubilized in DMSO.

### Cell lines

The ovarian cancer cell lines used in this study, their *TP53* status and histological subtype are listed in Table 1. All cell lines were sourced from the NICR authenticated cell bank and regularly tested for Mycoplasma. A2780, IGROV-1, OAW42 and CP70 were cultured in RPMI-1640 supplemented with 10% (v/v) FBS and 5% (v/v) penicillin/streptomycin. The CP70 cell line harbors a heterozygous *TP53* mutation (c.514 G->T, p.Val172Phe) [54]. The *MLH1*-corrected CP70+ cell line was grown in RPMI-1640 supplemented with 10% (v/v) FBS and Hygromycin B (200 μg/ml: Life Technologies, Inc.) [54]. This cell line has the heterozygous *TP53* mutation (c.514 G->T, p.Val172Phe). SKOV-3 and MDAH-2774 cell lines were cultured in DMEM supplemented with 10% and 5% (v/v) FBS and penicillin/streptomycin respectively. As information on the *TP53* status of SKOV-3 and IGROV-1 in the literature was contradictory, sequencing was performed. The results of PCR-based Sanger sequencing of *TP53* exon 5 and exon 4 confirmed the wild-type *TP53* status of the IGROV-1 cell line (Supplementary Figure 1). For the SKOV-3 cell line, frame shift deletion (c.265delC, p.Pro89fsX33) was confirmed in *TP53* exon 4 and no substitution mutation (c.179 A->G) was detected in *TP53* exon 5 (Supplementary Figure 2). The MDAH-2774 cells were confirmed to harbor a *TP53* mutation located in exon 8 (c.818G->A, p.Arg273His) [55].

### Growth inhibition assays and median-effect analysis

The GI_50_ values, the required concentrations of each compound leading to 50% growth inhibition, were determined by Sulforhodamine B (SRB) growth inhibition assays for drug exposure over 72 hours and the absorbance of the re-dissolved SRB protein stain was measured at 570 nm using a 96-well plate spectrophotometer (Spectramax 250 Molecular Devices) [56]. Growth curves were constructed using GraphPad Prism statistical analysis software version 5.04. For combination treatment of Nutlin-3 or RG7388 with rucaparib, the wild-type *TP53* cell lines were treated for 72 hours with each agent alone and in combination simultaneously at constant 1:1 ratios of 0.25x, 0.5x, 1x, 2x, and 4x their respective GI_50_ concentrations. Median-effect analysis was used to calculate Combination Index (CI) and Dose Reduction Index (DRI) values [57] using CalcuSyn software v2 (Biosoft, Cambridge, UK).

### Western blotting

Lysis buffer (12.5 ml Tris HCL, 2g SDS, 10 ml Glycerol, 67.5 ml Distilled Water) was used to harvest whole-cell lysates, followed by sonication. The concentration of protein in the cell lysates was estimated by using a bicinchoninic acid (BCA) assay. Novex® 4-20% Tris-Glycine 12-well polyacrylamide gradient gels (Invitrogen, UK) were used to separate proteins. The separated proteins were transferred by perpendicular electrophoresis to a nitrocellulose Hybond™ C membrane (Amersham, Buckinghamshire, UK). Monoclonal Mouse Anti-Human primary antibodies Actin 1:1000 (#: A4700, Sigma-Aldrich), MDM2 1:300 (#: OP46-100UG, Merck Millipore), p21^WAF1^ 1:100 (#: OP64, Calbiochem), PUMA 1:1000 (#dd716, Santa Cruz Biotechnology) and p53 1:500 (#: NCL-L-p53-DO7, Leica Microsystems Ltd.) were used. Secondary goat anti-mouse HRP-conjugated antibodies (#: P0447/P0448, Dako) were used at 1:1000. All antibodies were diluted in 5% milk/1XTBS-Tween (w/v). Enhanced chemiluminescence (GE Life Sciences) and X-ray film (Fujifilm) were used to visualize the proteins.

### Flow cytometry

Cells were treated with Nutlin-3, RG7388 and rucaparib alone and with combinations of Nutlin-3 or RG7388 with rucaparib simultaneously at constant 1:1 ratios of 0.5x and 1x their respective GI_50_ concentrations for 24, 48 and 72 hours. Harvested cells, both floating and adherent, were washed with PBS and resuspended in 500 μL PBS with 1mg/mL sodium citrate (Sigma, St Louis, MO), 100 μg/mL propidium iodide (Sigma), 200 μg/mL RNAse A (Sigma) and 0.3% Triton-X (Sigma). Samples were analyzed on a FACSCalibur™ flow cytometer using CellQuest Pro software (Becton Dickinson, Oxford, UK). Cell cycle distribution was determined using Cyflogic (CyFlo Ltd, Turku, Finland).

### Caspase 3/7 activity assay

Caspase 3/7 activity was measured using a Caspase- Glo 3/7 assay following the manufacturer’s instructions (Promega, Southampton, UK).

### Statistical analysis

All statistical tests presented were carried out using GraphPad Prism version 5.04 software. A p-value of <0.05 was considered to be statistically significant based on at least n=3 experimental repeats.

## SUPPLEMENTARY MATERIALS FIGURES AND TABLE


