# Correction: Combination treatment with rucaparib (Rubraca) and MDM2 inhibitors, Nutlin-3 and RG7388, has synergistic and dose reduction potential in ovarian cancer

**DOI:** 10.18632/oncotarget.28523

**Published:** 2024-02-22

**Authors:** Maryam Zanjirband, Nicola Curtin, Richard J. Edmondson, John Lunec

**Affiliations:** ^1^Northern Institute for Cancer Research, Newcastle University, Newcastle Upon Tyne NE2 4HH, United Kingdom; ^2^Faculty Institute for Cancer Sciences, University of Manchester, Manchester M13 9WL, United Kingdom

**This article has been corrected:** In Figure 5, the actin band in the A2780 column contains a partial duplicate of the same band in the IGROV-1 column. The corrected Figure 5, produced using the original data, is shown below. The authors declare that these corrections do not change the results or conclusions of this paper.

Original article: Oncotarget. 2017; 8:69779–69796. 69779-69796. https://doi.org/10.18632/oncotarget.19266

**Figure 5:**
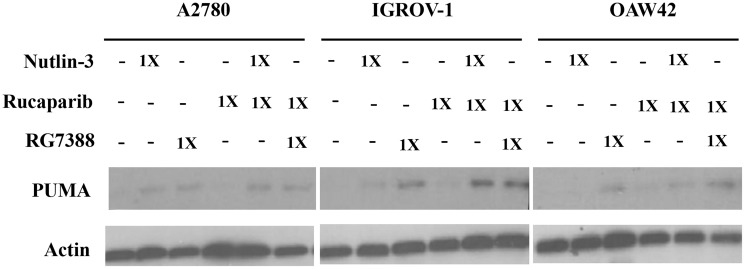
Combination of Nutlin-3/RG7388 with rucaparib increased upregulation of *TP53* downstream target, PUMA compared to rucaparib on its own but not compared to Nutlin-3/RG7388. Total levels of PUMA 24 hours after the commencement of treatment with Nutlin-3 and RG7388 alone, and in combination with rucaparib at constant 1:1 ratios of 1X their respective GI_50_ concentration analyzed by western blot in three wild-type *TP53* ovarian cancer cell lines.

