# Supplementary material for: Combination treatment with rucaparib (Rubraca) and MDM2 inhibitors, Nutlin-3 and RG7388, has synergistic and dose reduction potential in ovarian cancer
Source: Oncotarget. 2017 Jul 15;8(41):69779–96. doi: 10.18632/oncotarget.19266 (PMC5642516; doi:10.18632/oncotarget.19266)
Supplement: Supplementary file 1 [file oncotarget-08-69779-s001.pdf]

Combination treatment with rucaparib (Rubraca) and MDM2 inhibitors, Nutlin-3 and RG7388, has synergistic and dose reduction potential in ovarian cancer

SUPPLEMENTARY MATERIALS

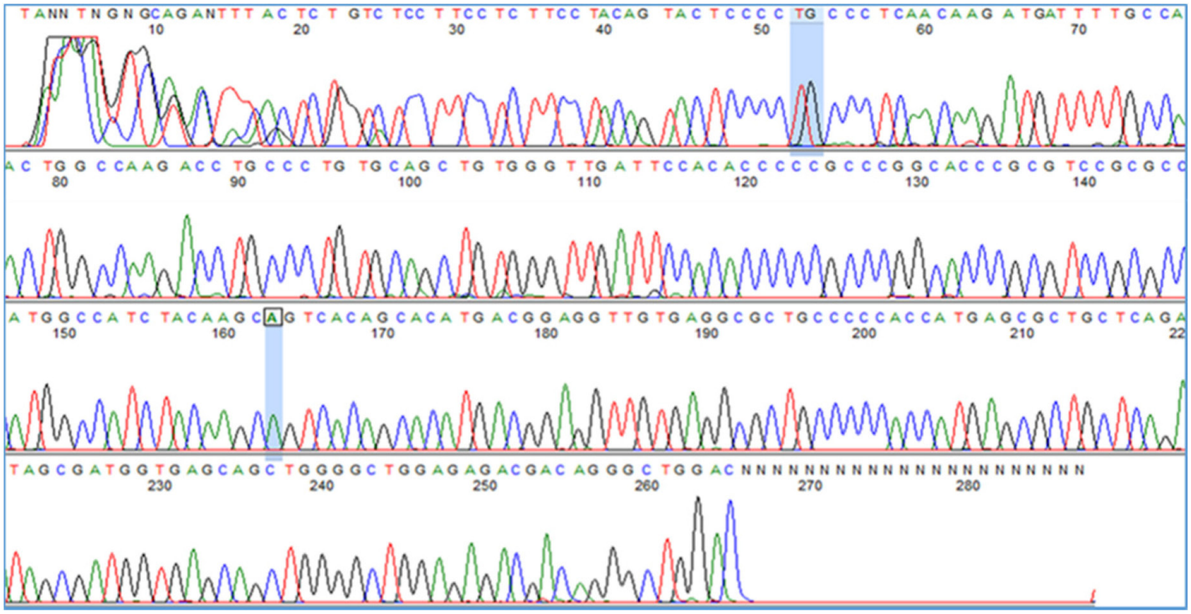

Supplementary Figure 1: Exon 5 DNA sequencing of the IGROV-1 cell line. Neither an insertion (c.267_268insC) nor a substitution mutation (c.377 A->G) was confirmed by Sanger sequencing.

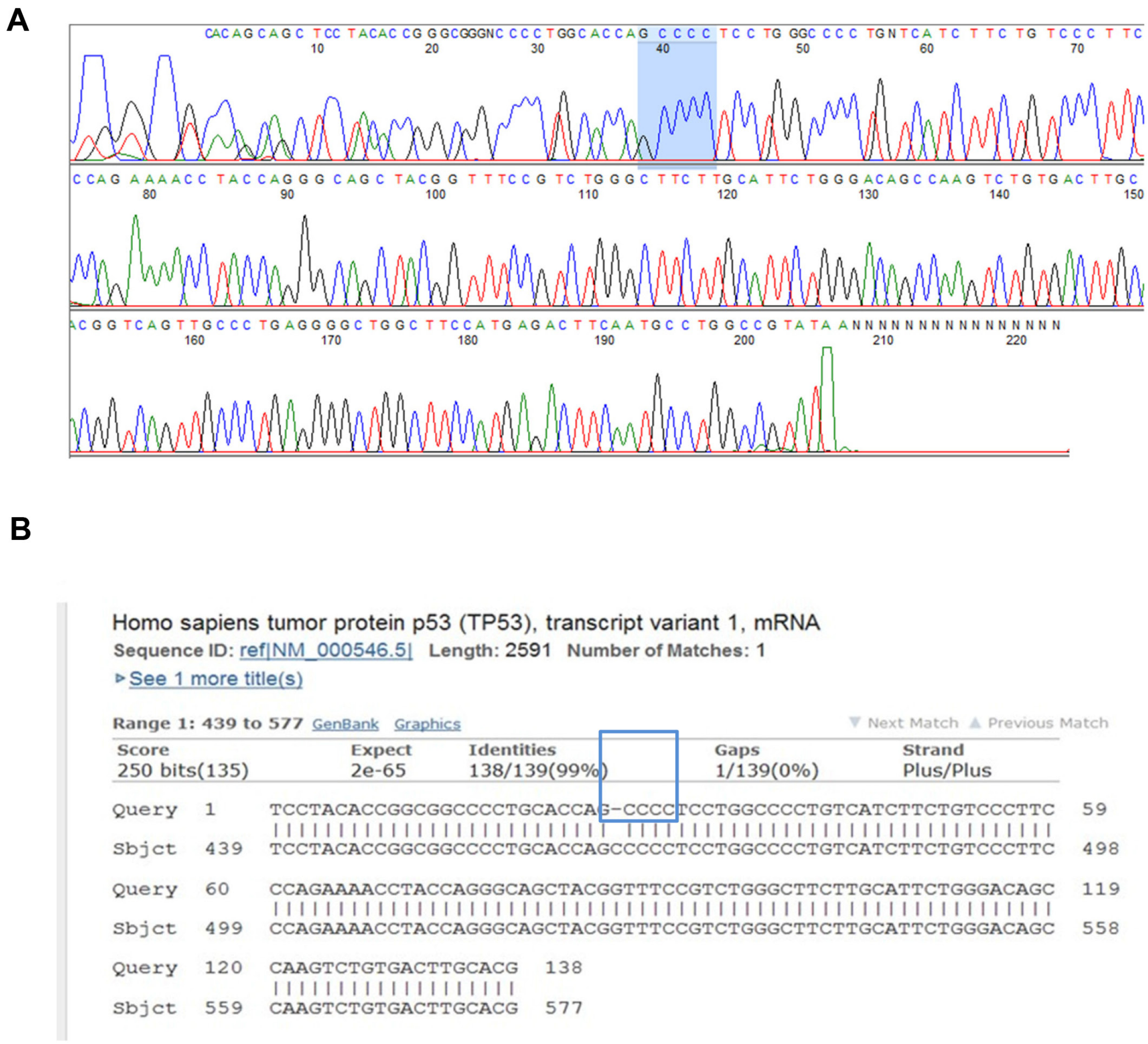

Supplementary Figure 2: Exon4 DNA sequencing of the SKOV-3 cell line. (A) Codon 89 of Exon 4 Cytosine deletion, frame shift (c.265delC, P.pro89fsX33). **(B)** The results of NCBI blast sequence alignment highlighting the deletion**.**

Supplemental Table 1: *BRCA1/2* gene status and BRCA-like signature in a panel of ovarian cancer cell lines used in this study. ND, Not determined. +/− = presence or absence of alteration. [ref]

Gene / Cell Line	Mutation								Amplification		Methylation		
	BRCA1	*BRCA2*	*ATM*	*ATR*	*CHK*	*RAD50*	*RAD51*	*PTEN*	*AURKA*	*EMCY*	*BRCA1*	*BRCA2*	*FANCF*
**A2780**	- [34]	- [34]	- [33]	- [33]	- [33]	- [33]	- [33]	+ [33 & 29]	- [29]	- [29]	- [34]	- [34 & 29]	- [35 & 36]
**IGROV-1**	+ [33 & 29]	- [34], + [33]	+ [33]	- [33]	- [33]	- [33]	+ [33]	+ [33]	ND	ND	- [34]	- [34]	- [36]
**OAW42**	- [34]	- [34]	- [33]	+ [33]	- [33]	- [33]	- [33]	- [33]	+ [29]	- [29]	- [34]	- [34 & 29]	+[36 & 37]
**CP70**	ND	ND	ND	ND	ND	ND	ND	ND	ND	ND	ND	ND	ND
***MLH1*-corrected CP70+**	ND	ND	ND	ND	ND	ND	ND	ND	ND	ND	ND	ND	ND
**MDAH-2774**	ND	ND	ND	ND	ND	ND	ND	ND	ND	ND	ND	ND	ND
**SKOV-3**	- [34]	-[34]	+ [33]	- [33]	- [33]	- [33]	- [33]	- [33]	- [29]	- [29]	- [34]	- [34 & 29]	- [35 & 36]
